# Mobility may impact attention abilities in healthy term or prematurely born children at 7-years of age: protocol for an intervention controlled trial

**DOI:** 10.1186/s12887-018-1229-1

**Published:** 2018-08-06

**Authors:** Hadrien Ceyte, Joëlle Rosenbaum, Isabelle Hamon, Maëlle Wirth, Sébastien Caudron, Jean-Michel Hascoët

**Affiliations:** 1 DevAH, Université de Lorraine, F-54000 Nancy, France; 2 Department of Neonatology, Maternité Régionale, CHRU, Université de Lorraine, F-54000 Nancy, France

**Keywords:** Premature infant, Children, Attention, Alerting, Orienting, Inhibition function, Mobility

## Abstract

**Background:**

Seven years of age is a milestone for learning basic knowledge that is strongly related to attention abilities such as Alerting, Orienting, and Inhibition function, allowing for appropriate adaptation to primary school. These attention abilities are also influenced by gestational age at birth in a complex manner, indicating an area of weakness in prematurely born children. Furthermore, recent studies suggest that allowing children to have freedom of movement during learning may improve their attention level and school performance. The purpose of the present study is to determine the influence of mobility on the attentional components that may impact learning abilities in children aged 7-years who were born at term and prematurely.

**Methods:**

This prospective, randomized, controlled trial will focus on psychometric testing of attentional abilities assessed with the Attention Network Test for Child (Child ANT) and involves a mixed measurement design. Forty-eight children aged 7-years, half of whom were premature at birth and in their expected grade without learning difficulties will be included after parental consent. They will be equipped with a head-mounted display in which the Child ANT will be presented. The association of different flankers and pre-cues will allow the measurement of the development level of Alerting, Orienting, and Inhibition function. The task will be composed of one experimental block of trials randomly performed per posture: seated, standing, or free.

**Discussion:**

This study will assess the contribution of mobility in specific attentional contexts that are usually present during fundamental learning in children. New pedagogical formats of teaching could consider these findings, and new pedagogical tools enabling free spontaneous child mobility might be designed. Moreover, a small percentage of children integrating into the educational system are born prematurely. These children, often considered immature and hyperactive, could benefit from educational innovations that enhance their attention abilities, thereby improving their adaptation to primary school.

**Trial registration:**

This trial is registered at ClinicalTrials.gov (NCT03125447).

## Background

Five to 7-years of age is a milestone in children’s development. At this age, they begin school and acquire the basics of fundamental learning such as reading, writing, and calculating. In a general way, these lessons are strongly related to attentional abilities and executive functions such as working memory and inhibitory control in children [1–8].

Since 1990, Posner’s work highlighted three different attentional networks that are thought to relate to the activation of different brain areas [9]. These cerebral networks are related to three components of attention: sustained attention or alerting (maintaining vigilance abilities), selective attention or orienting (ability to shift the attention), and inhibition function (ability to focus on one feature of a stimulus and ignore other interfering features). Fan et al. [10] developed an integrated Attention Network Test (ANT) based on a flanker task [11] in order to independently measure the efficiency of these three networks. This test was validated in adults, where alerting was induced by warning signals given prior to a target event, orienting facilitated by explicit spatial cues prior to a target event, and inhibition function evaluated by introducing incongruent flankers around the target. The adaptation of this test for children born at term at the ages of 4 to 10-years [12] showed an independence in the development of these three attentional systems. Alerting and orienting components may mature at up to 6-years of age then stabilize, while the inhibition function may improve up to the age of 7-years then remain stable after this age [12].

Little research has assessed the three attention components in children born prematurely. Studies suggest that prematurity may induce delays in maturation for the three attentional networks throughout the preschool years rather than lead to a persistent impairment [13, 14]. These attention components are influenced by age at assessment and gestational age at birth in a complex manner, indicating an area of weakness in children born prematurely [13]. On one hand, the risk for deficits in these attention components increases with decreasing gestational age. On the other hand, the development of these attention components might follow different developmental trajectories in children who were born preterm. For alerting, studies did not show any difference between children born at term versus those born preterm at the age of about 8-years [15, 16]. For orienting, the adult level may be reached at 8-years of age in children born prematurely [15]. A developmental delay of about one year has been observed between children born at term versus those born preterm [17–20]. Finally, data suggest that the inhibition function is still affected by prematurity at the age of 7-years [15–17] and up to 11-years of age in some children [21]. However, many different experimental assessments have been used to study the inhibition function (Tapping Test, Go No-Go Test, Stroop Color World test, Continuous Performance Test, Test Everyday Attention for Children, etc), which have failed to determine consensual and accurate developmental delays for this attentional component.

In general, the attention level of children is considered to decrease when they are moving. The poor attentional performance in those born prematurely as compared to children born at term is also attributed to an impulsivity [16]. Moreover, the urge for mobility is frequently observed in school age children and is often described as “hyperactivity” [22]. However, this is a loaded concept because it implies an attention deficit hyperactivity disorder (ADHD). This concept is one of the most extensively studied childhood psychiatric disorders and has a precise definition [23–25]. The core of ADHD-hyperactive symptoms are poor sustained attention, deficient impulse control (impulsivity), and excessive activity level [24, 26–29]. Thus, this qualification of “hyperactive” is excessive due to the absence of primary attention problems in many of these children, raising the fundamental question of the role of their apparent excessive mobility.

Extensive neuroimaging data highlight the interconnection between cognitive capacities and the sensorimotor state [30]. Human posture and/or mobility governs both neurophysiological arousal [31–33] and cognitive performance [34–38]. In healthy adults, Barra et al. [39] showed that increased body swaying related to imposed postures improved the alerting performance without modulating the orienting and inhibition function. Therefore, contrary to common thinking, mobility does not always seem to be a source of distraction leading to a lack of concentration. For instance, Janssen et al. [40] showed that the implementation of a moderate intensity physical activity break during the school day enhances attention levels, thereby improving school performance. Beyond this exercise-facilitated cognition, several studies suggest that children working in classrooms equipped with desks that allow standing and movement during class time led to significant improvement in their attention, executive control, and working memory [41–44].

### Aim of the study

The purpose of the present study is to determine the influence of mobility on the attention components that may impact learning in healthy children aged 7-years born at term or prematurely. We hypothesize that the absence of mobility constraints may improve alerting performance by increasing arousal in children born at term, without influencing orienting or inhibition function. We also speculate that considering mobility in children born prematurely might help improve some of their attention abilities.

## Methods and design

This prospective, randomized, controlled trial will focus on psychometric testing of the attention components and will involve a mixed measurement design. The study will take place in the Maternité Régionale of CHRU Nancy. It will be conducted in accordance with the Declaration of Helsinki. It was approved by the Comité de Protection des Personnes Sud-Est III Ethics Committee (2017–010 B) and registered in the clinicaltrial.gov registry (NCT 03125447). Because the participants will be children, the signed consent of their parents will be requested after they have received written information related to the study. The children will also be asked for their oral consent. Data collected will be analyzed anonymously.

### Inclusion and exclusion criteria

Children aged 7-years will be included in the study. Half will be children who were born prematurely and the other half will be children born at term. For the preterm group, children were born prematurely at or before 34 weeks gestation. They were born and cared for at our level III institution and involved in our routine follow-up program. At the time of the 7 years follow-up visit parents and child will be informed about the study and asked for participation. When they agree to participate an appointment will be taken for the study. For the term group, the children will be recruited using an information leaflet displayed at Lorraine University.

All children will have a clinical examination performed by trained pediatricians. General information on children’s health, socio-demographic data, behavioral problems, vision screening at the time of the test as well as perinatal information will be recorded. These features will be taken into account as potential confounding factors. All children with visual, cognitive, or motor disabilities that would prevent the realization of the test will be excluded. Also, infants with ADHD-inattentive problems will not be part of this study.

### Materials

The Child ANT [12] will be generated by the software, E-Prime (version 3.0 professional; Psychological Software Tools®, Sharpsburg PA, USA) and presented through a head-mounted display (Wear Video Headphones, The Way In®, Vuzix Corporation, New York, USA).

### Design and setting of the study

**Fig. 1:**
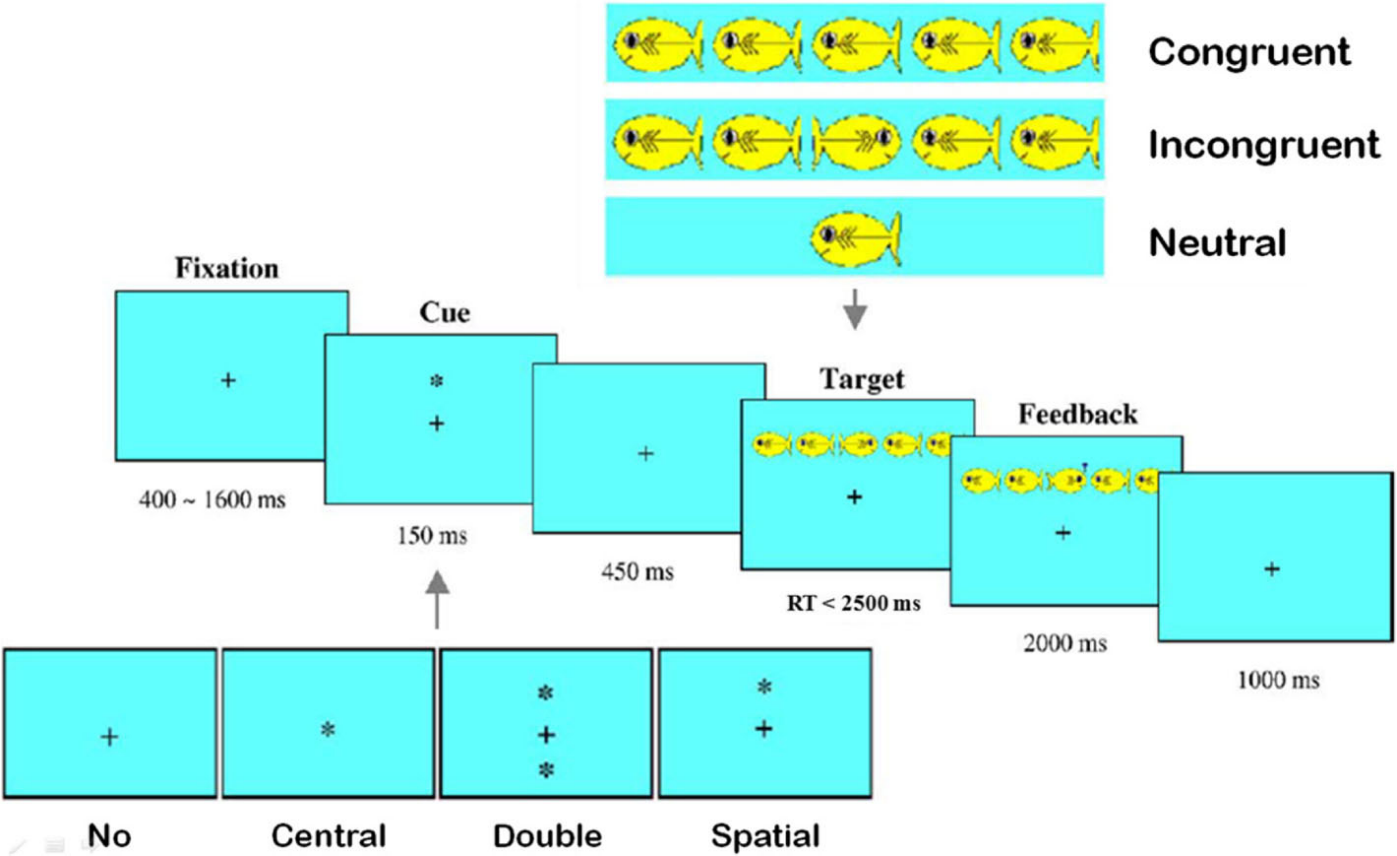
Schematic of the Attention Network Test for Child (Child ANT) adapted from Rueda et al [12]

The head-mounted display will be used to keep the distance between the eyes and the visual stimuli constant across 3 experimental conditions (seated, standing, and free position). The visual target stimulus consists of a yellow fish placed in the center of the visual blue background that is oriented toward the left or right side (Fig.1). This target will be presented either above or below a fixed cross and with or without flanker stimuli. The target will appear either alone (neutral condition) or in the center of a horizontal row of five yellow flanking fishes who will be oriented in the same direction (congruent condition) or in the opposite direction (incongruent condition). Children will be instructed to identify, as quickly and accurately as possible, the direction of the central fish by pressing the right or the left mouse button whatever the direction of the possible flanking fishes. The children will use their preferred hand. Each fish is subtended 1.6 degrees of visual angle and the contours of adjacent fish are separated by 0.2 degrees. The five fish are subtended a total of 8.8 degrees. The target will be presented about 1 degree above or below fixation.

Each target will be preceded by one of the following four warning cues (asterisk) conditions, as illustrated in Fig. 1: (1) no cue with only the fixation cross displayed; (2) a center cue presented at the location of the fixation cross; (3) a double cue, appearing simultaneously 1 degree above and 1 degree below the fixation cross then the target appears at the level of only one of these two cues; or (4) a spatial cue, appearing 1 degree above or 1 degree below the fixation cross, then the target appears at the location of the cue. Each trial will begin with a fixation period of random duration (400–1600 ms). After that fixation period, the warning cue will be presented for 100 ms and will be followed by another fixation period of 400 ms. subsequently, the target and flankers will appear simultaneously. They will be presented until the child responds. The maximal response time allowed will be 2500 ms. After the response, the target and flankers will disappear, and there will be a last fixation period of 3500 ms minus the response time (RT). Then, the next trial can begin.

To test the influence of mobility on the level of the three components of attention, we will ask the children to complete the experimental task in three random positions: (a) in a fixed seated position corresponding to a posture with very low mobility similar to the demand of sitting in a school environment; (b) standing in an upright position corresponding to the human’s reference posture, requiring real balance control due to the natural body sway; (c) in a free position where the children will be able to move and change their position whenever and as often as they want.

**Fig. 2:**
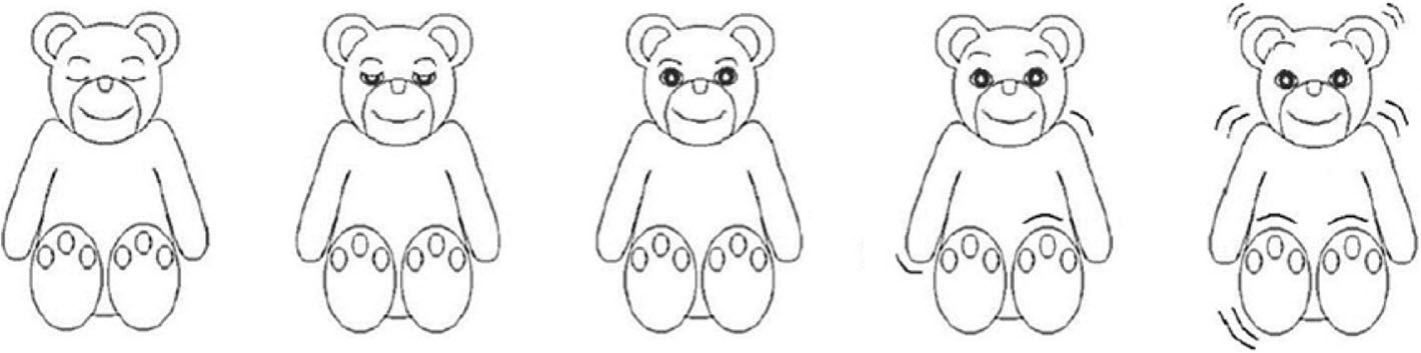
Teddy bears’ scale adapted from the Self-Assessment Manikin Scale

To check the children’s understanding of ANT, a 12-trial practice block, lasting less than 2 min, will be executed in the seated position. The children will receive feedback on their success. After this practice block, they will execute 48 trials in each position (with a 1-min 30-s break after 24 trials): 4 cue conditions × 2 target locations (up, down) × 2 target directions (left, right) × 3 flanker conditions (neutral, congruent, incongruent). The order of the trials will be randomized. Overall, each experimental block will last less than 3 min. Between each experimental block, the children will have a 3-min break. During these breaks, they will rate the subjective dimension of their arousal based on the adapted Self-Assessment Manikin scale [45]. They will point to one of five figures on a teddy bears’ scale (Fig.2), or between any two figures, which results in a 9-point rating scale. Overall, the experiment will last about 30 min.

### Data acquisition

To control for the position instructions, the experiment will be video recorded and an observation sheet completed for each experimental block.

During the Child ANT, the success and RT will be recorded for each trial. According to Fan et al. [10], the level of each attention components in each position will be computed from the RT difference of correct responses between pairs of specific trials. The alerting effect will be evaluated by subtracting the median RT of all double cue conditions for each child from the median RT of the no cue condition across the flanker conditions. The orienting effect will be evaluated by subtracting the median RT of all spatial cue conditions from the median RT of all center cue conditions across the flanker conditions. The inhibition function effect will be evaluated by subtracting the median RT of congruent flanking conditions from the median RT of incongruent flanking conditions across cue conditions.

### Statistical analyses

To determine the number of children to include, we relied upon Rueda et al. [12], showing a global sitting performance of an overall RT of 931 ± 42 ms in 6-years-old children born at term and 833 ± 123 ms in 7-years-old children born at term. Because children born prematurely are usually considered to have about a 1-year delay for learning abilities, we calculated that to demonstrate a catch-up related to the mobility condition, sitting being the reference, for each attention component, with an alpha risk of 0.00625 (Bonferroni correction for the number of tests) and a power of 0.80, 24 children would be needed in each group (Power and Precision™ V4, Biostat Inc., Englewood, NJ, USA 2001).

Thus, we will first compare the overall RT of the children born at term versus those born prematurely. Then, we will compare separately the mean scores (± standard deviation) obtained for each of the attention components. After having verified the required assumptions about data distributions (normality of attentional scores, homoscedasticity and sphericity), the level of each attention component will be analyzed by the means of three mixed analyses of variance, with position condition (seated, standing, free) as a within-subject factor and gestational age (children born preterm vs. children born at term) as a between-subject factor. For all analyses, post hoc tests will be conducted using Tukey’s honestly significant difference method when needed.

To evaluate the subjective arousal level of children between the positions, the Friedman test will be performed on each score on the Self-Assessment Manikin scale with position (seated, upright, and free) as a within-subject factor.

The statistical thresholds for significance will be set to 0.05 for the remaining analyses.

## Discussion

The consequences of child mobility during learning are a recurrent concern for parents and teachers. In general, a behavior with a high level of mobility is perceived as the expression of a lack of concentration, and consequently a lack of performance. This study will reassess the contribution of mobility expression in specific attentional contexts that are usually present during fundamental learning in children aged 7-years.

Numerous findings suggest that mobility is not always a source of distraction [39, 46–49]. The work of Stoffregen’s team [48, 49] suggests that during the accomplishment of a supra-postural cognitive task such as calculating or memorizing, the organism may generate a spontaneous body sway to facilitate the performance of the associated supra-postural task. The modulation of self-generated body motions may correspond to unintentional attempts to increase arousal. This would be enabled by the increase in physiological parameters leading to greater cerebral activation, hence facilitating information processing [40, 50–52]. This heuristic assumption results from the U-inverted model of Yerkes and Dodson [53], which proposes a progressive improvement in cognitive performance with a moderate increase in the arousal level until reaching a threshold of this energetic solicitation, when the cognitive performance progressively decreases.

Furthermore, the behavioral strategies in children, especially their mobility, should be considered in the analysis of their difficulties during class time. In other words, we speculate that the spontaneous mobility often observed in school children may reflect a behavioral strategy when he/she is engaged in learning activities with attentional overload. This possible reassessment of child mobility has potentially important implications for educational practices in order to facilitate the attentional performance in children. A new pedagogical format of teaching could be proposed, taking into account the child’s mobility. Also, new pedagogical tools that allow the child to have free mobility could be designed such as stand-biased school desks [41–44]. Simple environmental changes in classrooms could enhance children’s cognitive functioning, driving their cognitive development and impacting educational outcomes. This could significantly improve learning abilities in children who were born preterm. These children are known to have poor or delayed development levels of attention. From the outcomes of this trial, educational innovations may be developed to help improve the adaptation to primary school in vulnerable children.
